# Study on the relationship between sarcopenia and its components and anorexia in elderly maintenance haemodialysis patients

**DOI:** 10.1002/nop2.1149

**Published:** 2021-12-14

**Authors:** Chao Li, Lin Chen, Li He, Yingjun Zhang, Hui Chen, Yuan Liu, Sikai Tang, Haotian Zheng

**Affiliations:** ^1^ Hemodialysis center Department of Nephrology and National Clinical Research Center for Geriatrics West China Hospital Sichuan University Chengdu China; ^2^ West China School of Nursing Sichuan University Chengdu China; ^3^ Department of Clinical Nutrition West China Hospital Sichuan University Chengdu China

**Keywords:** anorexia, elderly, haemodialysis, influence factors, sarcopenia

## Abstract

**Aim:**

This study aimed to investigate the current situation of sarcopenia and anorexia of elderly maintenance haemodialysis patients and analyse the influencing factors.

**Design:**

A cross‐sectional design was used in this study.

**Methods:**

One hundred and twelve elderly patients on MHD in 3 haemodialysis centres in Sichuan, China, were selected. Sarcopenia was diagnosed according to the criteria of the Asian Working Group for Sarcopenia (AWGS). Anorexia was assessed with the *Functional Assessment of Anorexia Cachexia Therapy/Anorexia Cachexia Subscale (FAACT/ACS)*. The relationship between sarcopenia and anorexia was analysed by logistic regression.

**Results:**

The prevalences of sarcopenia and severe sarcopenia in elderly MHD patients were 52.7% and 39.3%, respectively, and the prevalence of anorexia was 25.9%. Severe sarcopenia was independently associated with anorexia, and weekly exercise frequency was independently associated with anorexia. The low SMI value and slow gait speed were strongly associated with anorexia. This study complied with the STROBE checklist.

## 1 INTRODUCTION

The global population is ageing, and the average age of patients with kidney disease is increasing. Surveys in Western countries showed that people over the age of 75 years have the highest incidence of end‐stage kidney disease (Evans et al., 2018; Saran et al., 2019). After chronic kidney disease progresses to end‐stage kidney disease, most patients choose to be treated with haemodialysis. The prevalence of sarcopenia in elderly patients on maintenance haemodialysis (MHD) is elevated due to the reduced muscle mass and physical activity caused by age and factors related to treatment (Lou et al., 2019). Previous studies have shown that the prevalence of sarcopenia in elderly MHD patients is 33.7%~37% (Giglio et al., 2018; Kim et al., 2014).

### 1.1 Background

Sarcopenia, which is characterized by a reduction in skeletal muscle, is a progressive and widespread condition that mainly manifests as an accelerated loss of muscle mass and function (Boaz et al., 2019). The main cause of sarcopenia in the older population on dialysis is malnutrition. Studies have reported that all elderly MHD patients have mild/moderate malnutrition (Abdulan et al., 2019). Malnutrition not only reduces the quality of life and survival rate of haemodialysis patients (Gencer et al., 2019) but is also an important cause of sarcopenia (Woo, 2017). Protein loss, weakness and anorexia caused by dialysis and ageing are all causes of insufficient nutrient intake and eventually lead to malnutrition (López‐Montes et al., 2020; Sabatino et al., 2018). Among these, anorexia is a key factor.

Anorexia is characterized by the loss of the subjective desire to ingest food. Anorexia is associated with a significantly higher risk of all‐cause mortality (Landi et al., 2012). As the body ages, the senses of smell and taste change. At the same time, there may also be phenomena such as tooth loss and reduced chewing ability, which leads to a reduction in food intake (Doty et al., 1984). In addition, delayed gastric emptying and changes in hunger hormone and insulin levels are also important factors that affect food consumption in elderly individuals (Brogna et al., 2006). The loss of appetite is more pronounced in patients with kidney disease on MHD due to the presence of uremic toxins, inflammation and hormone disorders. Previous studies have shown that the prevalence of anorexia in MHD patients is 17%–32.6% (Bossola et al., 2013; Molfino et al., 2016), and the prevalence of anorexia in the general older population is 25% (Landi et al., 2012). At present, no studies have been performed investigating anorexia in elderly MHD patients (Landi et al., 2013; Tsutsumimoto et al., 2020). The results of surveys in the older population have shown that sarcopenia is independently associated with anorexia. The older MHD population has higher risks of sarcopenia and anorexia than the general older population. The status of and correlation between sarcopenia and anorexia in this population deserves further investigation.

### 1.2 Aim and research questions

In this context, this study was performed to determine the prevalences of sarcopenia and anorexia through a survey of elderly MHD patients in China, analyse the relationship between anorexia and sarcopenia and its components and provide preliminary evidence that can be used to prevent and treat anorexia in elderly MHD patients.

Research questions:

1. How about the prevalence of sarcopenia in elderly haemodialysis patients?
2. How about the prevalence of anorexia in elderly haemodialysis patients?
3. Do sarcopenia and its components are related to anorexia in elderly haemodialysis patients?

## 2 MATERIALS AND METHODS

### 2.1 Participants

A cross‐sectional study was conducted with patients who received treatment from January 2020–December 2020 at the MHD centres of three general hospitals in Sichuan, China. The inclusion criteria were as follows: (a) age ≥ 60 years; (B) regular MHD for ≥3 months (2–3 times per week); (c) normal cognitive ability and ability to communicate verbally and in writing; and (d) willingness to participate in this study and sign an informed consent form. The exclusion criteria were as follows: (a) treatment with combined peritoneal dialysis; (b) intellectual disability, poor language comprehension, unstable mental state or severe mental illness; and (c) inability to walk. A total of 126 patients were screened, and 14 patients were excluded (pacemaker implantation [*N* = 3], death [*N* = 2], inability to walk [*N* = 5] and refusal to participate [*N* = 4]). Ultimately, 112 patients were included in the study. This study was approved by the ethics committee of our hospital. After all patients signed written informed consent forms, the members of the research group collected the relevant sociodemographic information from the patients and performed the necessary measurements.

### 2.2 Data collection

All data were collected at the dialysis centre. Skeletal muscle quality was measured by nutritionists in the research group. Standardized training was conducted with the members of the research group before the survey to ensure the consistency of their understanding of the scale items. The purpose and significance of this study were explained to the patients, and the patients were informed that their information would be kept confidential during the study. The patients voluntarily signed the informed consent form. The general information of the patients and the anorexia scale were filled out on the Questionnaire Star APP, which was accessed by scanning the QR code on a smartphone. For patients without smartphones and those who could not read, the researcher reads the items and filled them out as dictated by the patients.

### 2.3 Assessment of sarcopenia

Skeletal muscle quality was assessed with device bioelectrical impedance (BIA; model: In body S10). Skeletal muscle mass (SMM), body mass index (BMI) and body fat were measured. The skeletal muscle mass index (SMI) was calculated as follows: SMI = SMM/height^2^. Skeletal muscle strength was evaluated as handgrip strength (HGS). During the measurement, the test subject was standing. The patient's feet were naturally separated, and the arms were relaxed at the patient's side. The non‐ostomy hand (the dominant hand for intubated patients) was used to hold the grip strength metre. The displayed number was the grip strength value, which was measured 3 times per person. The largest value was recorded. Physical activity was evaluated by the subject's gait speed. A straight line distance of 6 metres was marked on the ground with tape. Barrier‐free space (0.6 m) was delineated at the start and end of the test area. The patients walked at a regular pace, while the examiner recorded how long it took them with a stopwatch. Each patient performed the test twice, and average speed of the two tests was recorded.

### 2.4 Diagnostic criteria for sarcopenia

Sarcopenia was diagnosed according to the newly revised recommendations of the 2019 Asian Working Group for Sarcopenia (AWGS) as follows (Chen et al., 2020): (1) low appendicular skeletal muscle mass (ASM), indicated by a SMI value <7.0 kg/m^2^ for males and <5.7 kg/m^2^ for females; (2) low muscle strength, defined as a HGS < 28 kg for males and <18 kg for females; and (3) low physical activity, defined as a walking speed <1.0 m/s. If (1) was met at the same time as (2) or (3), sarcopenia was diagnosed; if all three were met at the same time, severe sarcopenia was diagnosed.

### 2.5 Anorexia

Anorexia was assessed using the *Functional Assessment of Anorexia Cachexia Therapy/Anorexia Cachexia* Subscale (FAACT/ACS). The questionnaire consisted of 12 items, with responses recorded on a 5‐point scale ranging from 0–4 (not at all, a little bit, somewhat, quite a bit and very much), with a total possible score of 48. The higher the score was, the lower the risk of anorexia. In 2010, an expert consensus concluded that a FAACT/ACS score ≤24 points indicates a diagnosis of anorexia (Muscaritoli et al., 2010). However, Adriana et al. pointed out that such a cut‐off was based solely on the consensus opinion of experts rather than on the results of clinical trials. In their study, the threshold was increased to 30 points to avoid missing patients with anorexia (Arezzo di Trifiletti et al., 2013). In another investigation of anorexia in MHD patients, Alessio et al. diagnosed anorexia based on a FAACT/ACS score ≤ 30. The results suggested that the FAACT/ACS score had high sensitivity and specificity for the assessment of anorexia in MHD patients and was more closely associated with food intake than the other available assessment tools for anorexia (Molfino et al., 2016). Therefore, this study used a FAACT/ACS score ≤30 as the cut‐off for the diagnosis of anorexia. Cronbach's α, test–retest reliability and half‐time reliability were all >0.8, which indicated good reliability (Zhou et al., 2017).

### 2.6 Statistical analyses

All data were statistically analysed using SPSS 20.0. Continuous data are expressed as the means ± standard deviations or medians (interquartile ranges). Categorical data are expressed as percentages. Normally distributed continuous data were compared between two groups with *t* tests; non‐normally distributed continuous data were assessed with non‐parametric Wilcoxon rank‐sum tests. The classified data were tested by chi‐square test. Logistic regression analysis was used to evaluate the associations of sarcopenia and its three components with anorexia. The level of significance was set at 0.05.

### 2.7 Ethical principles

Approval for the original study was obtained from the Institutional Review Board and complied with the Strengthening the Reporting of Observational Studies in Epidemiology (STROBE) checklist (Supporting Information). For patients who were eligible and interested in taking part in this study, researchers explained the study in detail and took written informed consents.

## 3 RESULTS

### 3.1 Basic information

A total of 112 elderly MHD patients were included in this study, of whom 60.7% were males. Table 1 shows the average age was 70.04 ± 7.48 years. The primary disease was chronic nephritis in 20.5% of the patients (*N* = 23), hypertension in 33.1% (*N* = 39) and diabetes in 23.2% (*N* = 26). The average time on dialysis was 67 (28,116) months. A total of 10.7% (*N* = 12) of the patients exercised 1–2 times a week, 23.2% (*N* = 26) of the patients exercised 3–4 times a week, 43.8% (*N* = 49) of the patients exercised 5 times a week, and 22.3% (*N* = 22) of the patients did not exercise. According to the sarcopenia diagnostic criteria recommended by the AWGS in 2019, the prevalence rate of sarcopenia in this study was 52.7% (*N* = 59), and the prevalence rate of severe sarcopenia was 39.3% (*N* = 44). The prevalence of anorexia in elderly MHD patients was 25.9% (*N* = 29). See Table 1 for details.

**TABLE 1:** Patients characteristics

Characteristics	Total (*N* = 112)	With anorexia (*N* = 29)	Without anorexia (*N* = 83)	*p* value
Age (year)	70.04 ± 7.48	72.07 ± 7.18	69.33 ± 7.49	.089
Gender				
Male	68 (60.70)	14 (48.30)	54 (65.06)	.111
Female	44 (39.30)	15 (51.72)	29 (34.94)	
Dialysis duration (month)	67 (28,116)	70 (16,117)	71 (28,122)	.923
Reason for end‐stage kidney disease				
Chronic glomerular nephritis	23 (20.50)	10 (34.48)	13 (15.67)	.084
Hypertension	39 (33.10)	11 (37.93)	28 (33.73)	
Diabetes	26 (23.20)	5 (17.24)	21 (25.30)	
Other	24 (23.20)	3 (10.34)	21 (25.30)	
Movement (times per week)				
1–2	12 (10.70)	5 (17.24)	7 (8.43)	.025*
3–4	26 (23.20)	6 (20.69)	20 (24.10)	
≥5	49 (43.80)	7 (24.14)	42 (50.60)	
No movement	25 (22.30)	11 (37.93)	14 (16.87)	
Sarcopenia, *N* (%)				
Yes	59 (52.70)	21 (72.41)	38 (45.78)	.013*
No	53 (47.30)	8 (27.59)	45 (54.22)	
Severe sarcopenia				
Yes	44 (39.30)	18 (62.07)	26 (31.33)	.004*
No	68 (60.70)	11 (37.93)	57 (68.67)	
SMI, kg/m^2^	6.28 ± 1.06	5.81 ± 1.03	6.44 ± 1.03	.005*
Gait speed, m/s	0.84 ± 0.21	0.71 ± 0.23	0.89 ± 0.19	<.001*
Grip strength, kg	23.40 ± 7.65	20.91 ± 7.10	24.26 ± 7.70	.042*

Note

Bold values and * stands for *p* < .05.

### 3.2 Proportions of patients with sarcopenia and distribution of exercise frequency in the groups with and without anorexia

**FIGURE 1:**
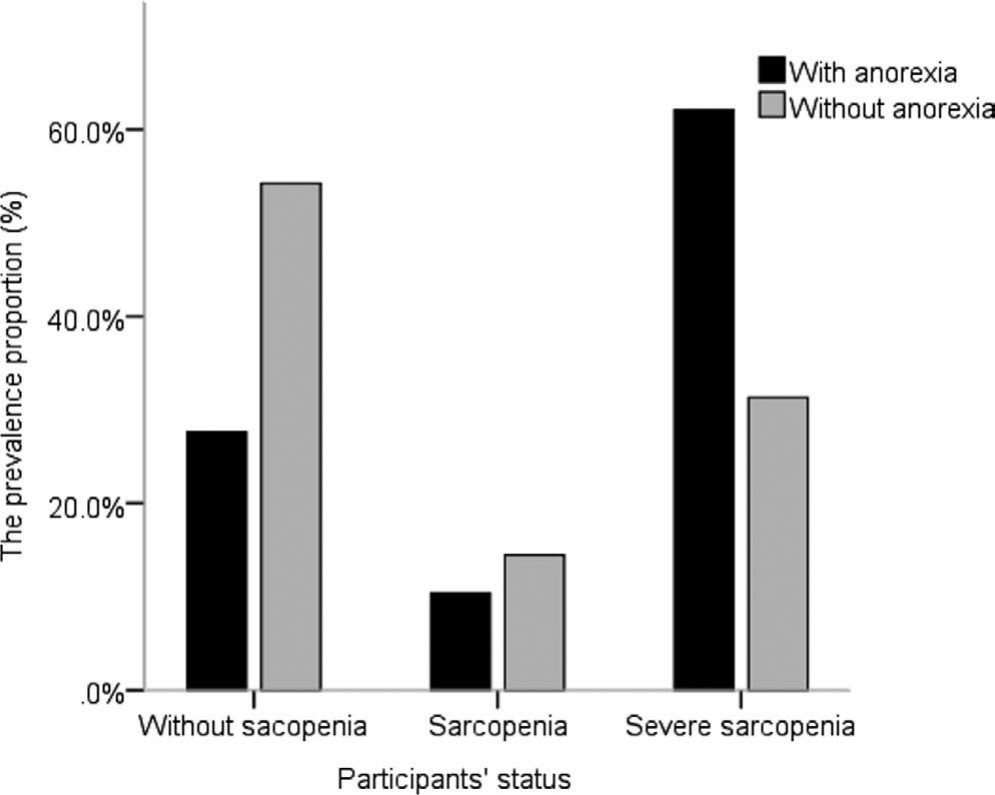
Prevalence of sarcopenia between elderly MHD patients with/without anorexia

Figure 1 shows that the proportions of patients without sarcopenia, with sarcopenia and with severe sarcopenia were 27.6%, 10.3% and 62.1% in the group with anorexia and 54.2%, 14.5% and 31.3% in the group without anorexia respectively.

**FIGURE 2:**
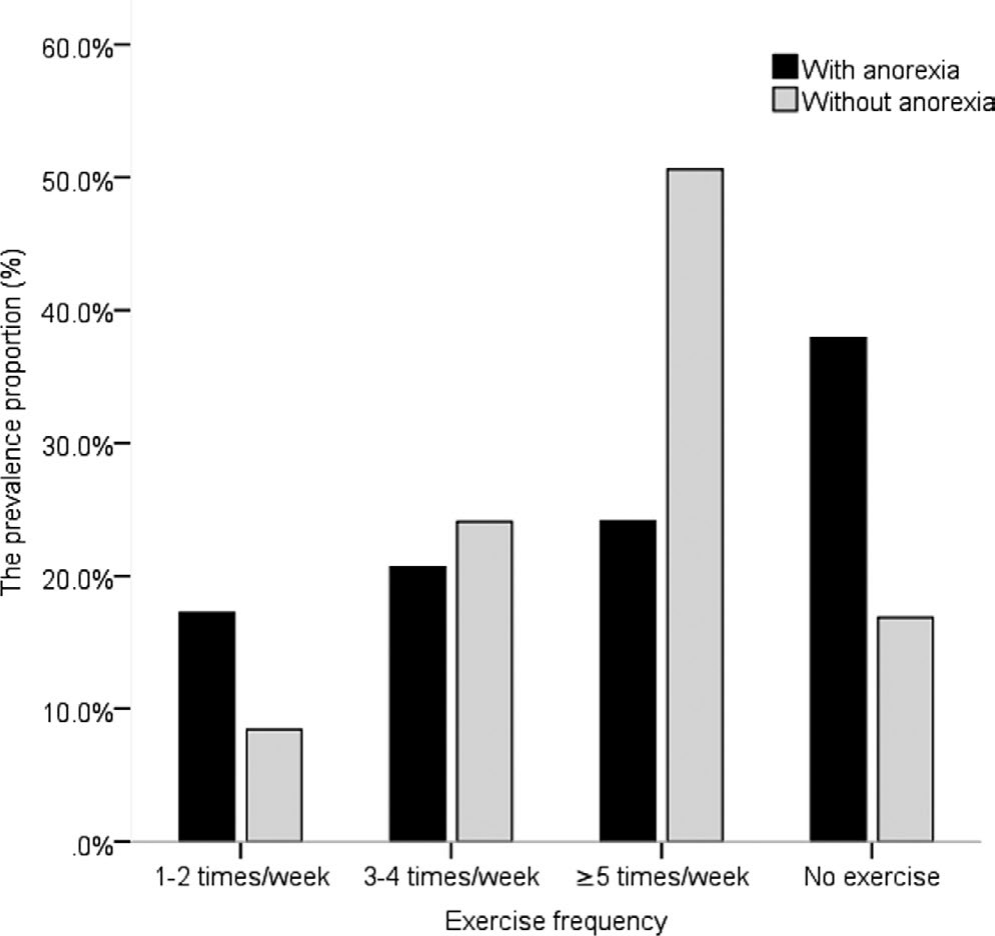
Prevalence of different exercise frequency between elderly MHD patients with/without anorexia

The group with anorexia had a larger proportion of patients with severe sarcopenia than the group without anorexia (*p* < .05). Figure 2 shows that the proportions of patients who exercised 1–2 times/week, 3–4 times/week, ≥5 times/week and 0 times/week were 17.2%, 29.7%, 24.1% and 37.9% in the group with anorexia and 8.4%, 24.1%, 50.6% and 16.9% in the group without anorexia respectively. The group without anorexia had a larger proportion of patients who exercised more than 5 times per week than the group with anorexia (*p* < .05).

### 3.3 Association between sarcopenia and anorexia in elderly haemodialysis patients

Table 2 shows that sarcopenia, severe sarcopenia and the weekly exercise frequency were associated with anorexia in univariate analyses. Severe sarcopenia (OR, 0.29 [95% CI: 0.09, 0.97]) and exercise frequency (OR, 3.77 [95% CI: 1.03, 13.83]) were independently related to anorexia after adjustment for age, sex, primary disease and dialysis duration.

**TABLE 2:** Association between Sarcopenia and anorexia on elderly MHD patients

Variable	Univariate analysis		Multivariate analysis	
	OR (95% CI)	*p* value	OR (95% CI)	*p* value
The degree of sarcopenia				
No sarcopenia	Reference			
Sarcopenia	0.71 (0.16, 3.10)	.650	0.70 (0.15, 3.36)	.658
Severe sarcopenia	0.26 (0.10, 0.67)	.006	0.29 (0.09, 0.97)	.045*
Exercise (times per week)				
No exercise	Reference			
1–2	1.10 (0.27, 4.43)	.893	0.88 (0.16, 4.80)	.882
3–4	2.62 (0.78, 8.75)	.118	1.87 (0.47, 7.35)	.372
≥5	4.71 (1.53, 14.51)	.007	3.77 (1.03, 13.83)	.046*

Note

Multivariate analysis: adjusted for age, sex, reason for end‐stage kidney disease, dialysis duration, and movement. *p < .05

### 3.4 Association between the components of sarcopenia and anorexia in elderly haemodialysis patients

Table 3 shows that the SMI, gait speed and grip strength were related to anorexia in the single‐factor analyses. After adjusting for age, sex, primary disease, dialysis duration and exercise frequency, the results showed that the SMI (OR, 2.52 [95% CI: 1.13, 5.64]) and gait speed (OR, 1.08 [95% CI: 1.02, 1.14]) were independently related to anorexia.

**TABLE 3:** Association between Sarcopenia's Components and anorexia on elderly MHD patients

Variable	Univariate analysis		Multivariate analysis	
	OR (95% CI)	*p* value	OR (95% CI)	*p* value
SMI	1.81 (1.18, 2.80)	.007	2.52 (1.13, 5.64)	.025*
Gait speed, m/60 s	1.08 (1.03, 1.118)	<.001	1.08 (1.02, 1.14)	.005*
Grip strength, kg	1.07 (1.00, 1.13)	.045	0.94 (0.85, 1.04)	.209

Note

Multivariate analysis: adjusted for age, sex, reason for end‐stage kidney disease, dialysis duration, and movement. *p < .05.

## 4 DISCUSSION

The results of this study show that sarcopenia and anorexia are relatively more common in elderly MHD patients. Anorexia is independently related to severe sarcopenia and the weekly frequency of exercise. In addition, the SMI and gait speed are independently related to anorexia.

In this survey, the prevalence of sarcopenia in the older MHD population was 52.7%, and the prevalence of severe sarcopenia was 39%. The prevalence of sarcopenia in the older MHD population surveyed by Kim et al. was 33.7%(Kim et al., 2014), and Cheng et al.'s survey of MHD patients of all ages showed that the prevalence of severe sarcopenia was 30.7% (Cheng et al., 2020), which was lower than that found in this study. A possible reason is that the mean age of the patients in this survey was 70.04 ± 7.48 years, which was older than the 63.9 ± 10.0 years and 60.9 ± 13.2 years reported in Kim's and Cheng's surveys. Research has shown that advanced age is a risk factor for sarcopenia (Mori et al., 2019). As people age, their muscle strength and quality gradually decrease; therefore, older people are relatively more probably to develop sarcopenia. The prevalence of sarcopenia in the older MHD patients surveyed by Giglio was 37% (Giglio et al., 2018), which is still lower than that reported in this study. This may be related to the differences in the diagnostic criteria for sarcopenia, as the criteria recommended by the EWGSOP in 2010 were used in Giglio's study, while those recommended by the ASWG in 2019 were used in this study. In addition, Giglio surveyed a South American population, while this study surveyed an Asian population. Studies have shown that the prevalence of sarcopenia is higher in Asians with MHD than in Black and white populations (Yoowannakul et al., 2018). Two surveys of sarcopenia were performed in the general population older than 60 years in Brazil, and the prevalence rates were 55.8% and 45.8%; one survey reported a prevalence of severe sarcopenia of 6.9% (de Amorim et al., 2019; Confortin et al., 2018). The prevalences of sarcopenia in those two studies were similar to the results of this study, but the prevalence of severe sarcopenia was higher in this study. This may be related to factors that accelerate the development of sarcopenia, such as nutritional deficiencies, acidosis, vitamin D deficiency and inflammation caused by MHD (Domanski & Ciechanowski, 2012). The combined effects of advanced age and dialysis lead to a more pronounced decrease in skeletal muscle mass. Therefore, it can be inferred that patients who are elderly and on MHD are more probably to develop sarcopenia and severe sarcopenia than the general elderly population and nonelderly MHD patients. In clinical practice, nursing staffs should strengthen the screening and recognition of sarcopenia in elderly patients with MHD, and effective interventions should be implemented as soon as possible to reduce the harm caused by sarcopenia in this population.

The prevalence of anorexia in elderly MHD patients in this survey was 25.9%. A validation study of appetite assessment tools in MHD patients showed that the prevalence of anorexia diagnosed based on FAACT/ACS was 17% (Molfino et al., 2016), which is lower than that in this study. Alessio performed a study to verify appetite assessment tools in MHD patients and found that the prevalence of anorexia diagnosed with FAACT/ACS was 17%, which is lower than that in this study. This may be related to the average age of the patients in this survey of 70.04 ± 7.48 years, which is older than the average age of 60.2 ± 14.6 years in the study by Alessio. Anorexia is a common health problem in elderly patients. As people age, appetite loss and insufficient food intake become more obvious (Visvanathan, 2015). Related reports have shown that MHD patients experience a gradual decrease in appetite with age (Katkov et al., 2018). Anorexia has not been studied in elderly MHD patients, but studies in other elderly populations have shown that the prevalence of anorexia is between 9.8%–25% (Donini et al., 2011; Landi et al., 2012; Tsutsumimoto et al., 2020). The populations investigated in those three studies were healthy, community‐dwelling elderly individuals, elderly individuals enrolled in home care programmes and elderly individuals in rehabilitation wards with acute illnesses. The prevalence of anorexia in this study was higher than those in previous studies. The reason may be related to factors specific to the population undergoing MHD, such as the presence of uremic toxins, inflammation and hormones, which are involved in the development of anorexia (Bergstrom, 1999; Carrero et al., 2008). Therefore, elderly individuals who are undergoing MHD are more probably to develop anorexia than the general older population.

This study showed that anorexia was independently associated with severe sarcopenia in elderly patients on MHD. Sarcopenia and anorexia have similar causes, and both are geriatric syndromes. In previous studies, sarcopenia was found to be independently associated with anorexia of ageing in elderly patients, which is similar to the results of this study (Landi et al., 2013; Tsutsumimoto et al., 2020). Anorexia of ageing refers to the loss of appetite caused by various physiological and pathological causes associated with old age, which results in changes in nutritional status in the older population (Landi et al., 2017). Loss of appetite means a low intake of nutrients. In the study by Jyvakorpi, it was mentioned that even in the group of elderly individuals with normal nutritional status, a large proportion had low energy, low protein and low micronutrient intake levels (Jyvakorpi et al., 2016). A low intake of nutrients is the main cause of sarcopenia (Bonnefoy et al., 2015; Giglio et al., 2018). Therefore, in subsequent studies, we can continue to explore whether improving anorexia in elderly MHD patients has an effect on sarcopenia. In clinical practice, nursing staffs can carry out relevant dietary education through the evaluation of patients' nutritional status, so as to improve patients' dietary compliance.

This study adjusted for factors such as age, sex, primary disease, dialysis duration and weekly exercise frequency and found that grip strength was not correlated with anorexia, while the SMI and gait speed were independently correlated with anorexia. The sarcopenia diagnostic criteria recommended by the ASWG are based on three indicators: SMI, gait speed and grip strength. Cheng proposed that the measurement of gait speed can effectively be used to screen for impairment of activities of daily living in MHD patients (Cheng et al., 2020). The study by Kittiskulnam showed that gait speed and grip strength were associated with mortality in MHD patients (Kittiskulnam et al., 2017). The SMI plays an important role the assessment of whether elderly individuals need nutritional support or increased physical activity (Zhang et al., 2019) and can also be used as an indicator of the improvement in tongue strength in patients with dysphagia (Nakazawa et al., 2020). A change in gait speed is correlated with reduced quality of life and independence in the older population (Malafarina et al., 2013) and can be used to identify vulnerable individuals (Kyrdalen et al., 2019). Interestingly, this study also showed that anorexia was independently associated with the frequency of exercise per week. Patients who exercised more than 5 times per week were less probably to develop anorexia. Reduced physical activity and poor mobility can easily lead to an impaired ability to engage in routine daily activities, which can lead to difficulty eating independently (Landi et al., 2017). Therefore, the prevalence of anorexia may be lower in patients who can exercise independently. Previous studies have suggested that in elderly individuals, exercise can effectively improve appetite, increase muscle strength, improve SMM and reduce the risks of sarcopenia and muscle weakness (Davis et al., 2009; Sanford, 2017). Therefore, in elderly MHD patients, anorexia can be prevented by increasing the frequency of resistance training, which can prevent the loss of muscle strength and reduce the occurrence of sarcopenia. During dialysis, elderly MHD patients can exercise on bed properly with the guidance of nursing staffs. For example, low‐intensity leg exercises include leg lifts, quadriceps femoris contraction and knee flexion. However, in this study, the exercise mode of patients was relatively simple (only walking), and further research is needed to determine the associations of other types of exercise with anorexia.

## 5 CONCLUSION

In conclusion, sarcopenia and anorexia are common in elderly patients on MHD. Patients with severe sarcopenia are more probably to suffer from anorexia. Elderly MHD patients with low SMI and high gait speed were more probably to suffer from anorexia, and those who did not exercise were more probably to suffer from anorexia than those who exercised more than 5 times per week. To determine whether increasing physical activity, especially resistance training, can reduce the incidences of sarcopenia and anorexia in elderly MHD patients, prospective interventional studies are needed.

### 5.1 Relevance to clinical practice

Sarcopenia and anorexia are common in elderly MHD patients. Patients with sarcopenia may be more prone to anorexia. Elderly MHD patients with anorexia are related to severe sarcopenia and the components of sarcopenia. Nursing staffs can strengthen the prevention and treatment of sarcopenia and anorexia. Nursing staffs should also consider whether to prevent anorexia by strengthening exercise management in elderly MHD patients.

### 5.2 Limitation

The limitations of this study are as follows. First, this study was a cross‐sectional study; therefore, causal relationships between sarcopenia and anorexia could not be determined. Second, convenience sampling was used in this study, which meant that the self‐management of the primary disease was unknown for the participants at the three dialysis centres. This may have influenced sarcopenia and the prevalence of anorexia. Third, during the information collection process, primary disease and weekly exercise frequency were reported by the patients, and memory bias or errors could have affected the results of the statistical analysis. Fourth, BIA was used to evaluate skeletal muscle quality, which is more susceptible to errors caused by oedema and other conditions than dual‐energy X‐ray absorptiometry. Therefore, all BIA measurements were completed after the dialysis session to minimize errors caused by oedema. Finally, there is no standardized method of assessing anorexia, and the use of the FAACT/ACS scale for the assessment of anorexia in elderly MHD patients needs further research.

## CONFLICT OF INTEREST

The authors declare that they have no conflict of interest.

## AUTHOR CONTRIBUTIONS

All authors have agreed on the final version and meet at least one of the following criteria (recommended by the ICMJE [http://www.icmje.org/recommendations/]): Substantial contributions to the conception or design of the work; or the acquisition, analysis, or interpretation of data for the work; Drafting the work or revising it critically for important intellectual content; Final approval of the version to be published; Agreement to be accountable for all aspects of the work in ensuring that questions related to the accuracy or integrity of any part of the work are appropriately investigated and resolved.

## Supporting information

Supplementary Material

## Data Availability

The raw/processed data required to reproduce these findings cannot be shared at this time as the data also form part of an ongoing study.
